# The Effectiveness of Percutaneous Vertebroplasty Is Determined by the Patient-Specific Bone Condition and the Treatment Strategy

**DOI:** 10.1371/journal.pone.0151680

**Published:** 2016-04-21

**Authors:** René P. Widmer Soyka, Benedikt Helgason, Javad Hazrati Marangalou, Joop P. van den Bergh, Bert van Rietbergen, Stephen J. Ferguson

**Affiliations:** 1 Institute for Biomechanics, ETH Zürich, Zürich, Switzerland; 2 Orthopaedic Biomechanics, Department of Biomedical Engineering, Eindhoven University of Technology, Eindhoven, The Netherlands; 3 Department of Internal Medicine, Maastricht University Medical Center, Maastricht, The Netherlands; 4 Department of Internal Medicine, Viecuri Medical Center, Venlo, The Netherlands; Le Fe Health Research Institute, SPAIN

## Abstract

**Purpose:**

Vertebral fragility fractures are often treated by injecting bone cement into the collapsed vertebral bodies (vertebroplasty). The mechanisms by which vertebroplasty induces pain relief are not completely understood yet and recent debates cast doubt over the outcome of the procedure. The controversy is intensified by inconsistent results of randomized clinical trials and biomechanical studies that have investigated the effectiveness or the change in biomechanical response due to the reinforcement. The purpose of this study was to evaluate the effectiveness of vertebroplasty, by varying the relevant treatment parameters and (a) computationally predicting the improvement of the fracture risk depending on the chosen treatment strategy, and (b) identifying the determinants of a successful treatment.

**Methods:**

A Finite Element model with a patient-specific failure criterion and direct simulation of PMMA infiltration in four lumbar vertebrae was used to assess the condition of the bone under compressive load before and after the virtual treatment, simulating in a total of 12000 virtual treatments.

**Results:**

The results showed that vertebroplasty is capable of reducing the fracture risk by magnitudes, but can also have a detrimental effect. Effectiveness was strongly influenced by interactions between local bone quality, cement volume and injection location. However, only a moderate number of the investigated treatment strategies were able to achieve the necessary improvement for preventing a fracture.

**Conclusions:**

We conclude that the effectiveness of vertebroplasty is sensitive to the patient’s condition and the treatment strategy.

## Introduction

Osteoporosis is a common disease that manifests itself in fractures occurring at different anatomical sites, e.g. at the spine, the hip, or the wrist, with consequent morbidity and mortality [1–3]. Augmentation through the injection of a reinforcing biomaterial (percutaneous vertebroplasty, PVP), is a popular, minimally invasive intervention for the stabilization of fractured vertebral bodies. The utility and effectiveness of PVP is most often evaluated on the basis of three measures: economic value (i.e. treatment and opportunity costs vs. effect, cost-effectiveness), short- and long-term pain relief and the incidence of new fractures either at the treated, adjacent or remote levels. Cost-effectiveness determines whether, given a cost level that society is willing to spend per quality-adjusted life-year (QALY), PVP is an acceptable treatment strategy or not. Hence, cost-effectiveness depends directly on the other two measures. Both were in the past and more recently addressed by numerous studies, with controversial results and conclusions on the utility and effectiveness of PVP [4]. One potential cause of the discrepancy might be the fact that these studies were driven by different objective targets. For example, immediate pain relief in a short period of time is important for elderly patients while a sustainable treatment outcome is beneficial for younger patients with an acute vertebral fracture. Two studies [5, 6] with a sham control intervention have reported clinical outcomes at one and six months, respectively, in patients with osteoporotic compression fractures up to one year old and cast doubts on the effectiveness of PVP.

In both studies, patients were randomized to undergo either a PVP or a sham procedure. Buchbinder et al. [5] concluded that”similar improvements were seen in both groups with respect to pain at night and at rest, physical functioning, quality of life, and perceived improvement”. The study of Kallmes et al. [6] found similar results and showed that improved disability and pain scores were noted immediately following both procedures however, a trend towards a higher rate of clinically meaningful improvement in pain (30% decrease from the baseline) was observable in the PVP group. These results have been met with some disbelief among physicians treating patients with vertebral compression fractures, and concerns have been raised on the design of both studies (ethical concerns, small sample size, crossover between the groups, i.e. patients were able to guess they were being treated with the sham procedure, and the sham procedure could itself have promoted pain relief, inclusion of patients with subacute and chronic fractures instead of acute fractures) [7–11]. The psychological effect of care and daily attention are believed to account for the decrease of the visual analogue score in conservative treatment groups during the first week of the treatment. Robinson and Olerud [4] concluded that PVP is not better than placebo and PVP cannot be recommended as a standard treatment for vertebral compression fractures. Conflicting with these results are other studies showing the utility and cost-effectiveness [11–13] of PVP, demonstrating both short-term and sustainable pain relief [14–22] and statistically significant differences in life expectancy in favour of PVP [23].

Several biomechanical studies have investigated the consequences of the augmentation on adjacent or remote, non-augmented levels. Most of them [24–32] concluded that new fractures might be the consequence of an adjacent rigid reinforcement. However, new compression fractures after the treatment could also be the consequence of the natural progression of the disease, since initial fractures are also a strong predictor of further vertebral fractures [33]. Inconsistently, clinical studies [17, 20] reported that the incidence of new fractures was not different after PVP, compared with conservative treatment after one year of follow-up. The major limitations of the biomechanical studies that could explain these divergent conclusions were the limited number of specimens and/or experimental trials (a problem generally associated with cadaveric testing) [25, 26, 28–32, 34–36] and Finite Element (FE) models that were limited to the study of a single case [26, 29, 30], constructed on the basis of non-realistic, predefined cement distributions [24, 31, 37–39], cement distributions reconstructed from patient radiographs [40] or peripheral quantitative computed tomography (pQCT) scans of human cadaveric specimens [41] with potentially suboptimal cement placement and/or filling strategy. Furthermore, the complex composite properties of cement-augmented bone have been neglected. A fundamental limitation of such biomechanical studies is the evaluation of physical units (endplate deflection [24, 30], failure load [28], maximum principal strain [30], pressure [26, 29, 30], stresses [29, 37, 39, 41], stiffness [30, 31] or strength [34, 37, 38] that form the basis for the inference as to whether PVP induces adjacent fractures or is efficient from the biomechanical perspective. It has been shown that absolute values alone, e.g. the bone mineral density (BMD), are often suboptimal predictors of osteoporotic fractures [42–44], since the distributions of those values of the different populations can overlap and do not allow sharp discrimination between the populations. The establishment of FE models from computed tomography (CT) data has been successful to investigate patient specific bone strength in-vitro and these perform better in the explanation of failure load variability among different subjects [42, 45]. These models are considered capable of capturing most of the tissue properties which contribute to bone strength and simulate the mechanical consequence of general external boundary conditions. Therefore, an FE model with the material properties derived from a clinical CT scan and an appropriate failure criterion is feasible to reflect and assess the mechanical condition or stability of the individual patient’s vertebrae prior to treatment, i.e. (a) how close a given vertebra is to failure or (b) the amount of mechanical stabilization needed to prevent the failure. Extending the FE model and failure criterion with a constitutive law, which describes accurately the response of the augmented bone cement composite, allows subsequently to locally predict the increase or decrease of fracture risk state induced by PVP and whether the positive changes go far enough to prevent the subsequent fracture of the bone. Furthermore, until now, no evidence has been provided as to which extent cement type, cement volume, cannula placement and other treatment parameters affect the aforementioned loading pattern in the spine and the final outcome of the treatment. This study aims therefore to systematically investigate potential PVP strategies in an in-silico environment that allows for the creation of large set of virtually performed PVP treatment cases. Comparative values are used to measure the change of the local factor of fracture risk and therefore give an indication, (a) if vertebroplasty is an efficient treatment from the biomechanical point of view, and (b) what the important determinants of a positive outcome are.

## Methods

### Specimens and μCT data

Four human vertebra (Table 1), provided by the International Institute for the Advancement of Medicine (IIAM, 175 May Street, Edison, NJ 08837, http://www.iiam.org/), from two male donors were used in this study. The specimens were scanned using a high resolution computed tomography scanner (XtremeCT, Scanco Medical AG, Brüttisellen, Switzerland) at the Maastricht University Medical Center. The scans, performed in air, were taken at a nominal isotropic resolution of 41 μm and segmented using a global threshold. A mesh morphing technique [46] was applied to map a template finite element (FE) mesh of a vertebra with 51119 elements onto the segmented CT data for each of the specimens. Following, for each element in the mesh the density, trabecular spacing (Tb.Sp) and fabric were calculated for a 4 mm spherical region centered at the element centroid from the original high-resolution images [47].

**Table 1:** Specimen data. The volumetric bone mineral density (vBMD) was estimated based on the bone mineral content and volume of the elements of the FE meshes that did not have any nodes on the surface of the models in order not to include any cortical shell in the vBMD calculations.

Specimen	Level	Donor	Age (years)	Gender	vBMD (g/cm^3^)
**1**	L1	I	88	M	0.187
**2**	T12	I	88	M	0.360
**3**	L1	II	70	M	0.113
**4**	T12	II	70	M	0.187

The meshes were then subsequently used to carry out a sequence of mechanical and flow FE simulations as described in detail below.

### FE simulations—pre-augmentation fracture risk

**Fig 1:**
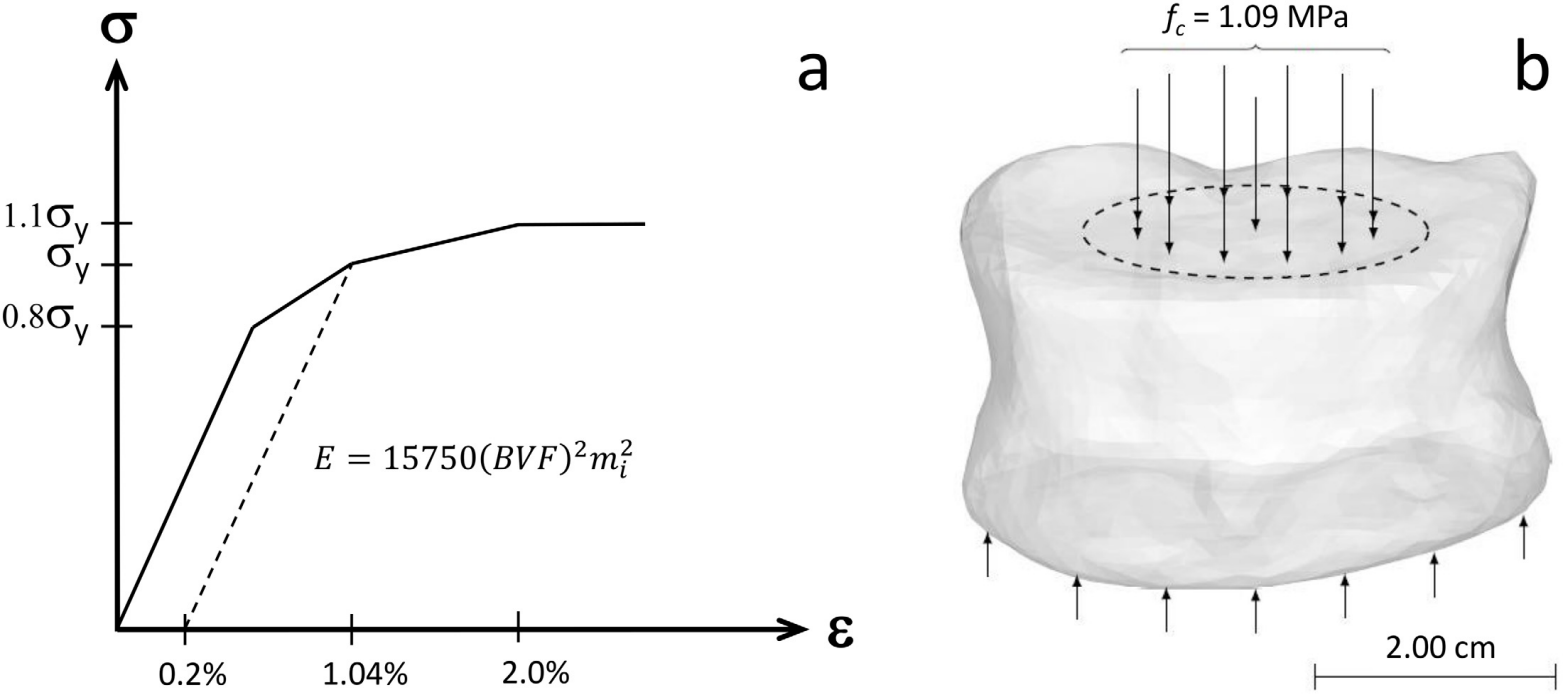
Finite Element Analysis setup. (a) Principal material direction stress-strain response mapped to the elements of the FE meshes. The *m*_*i*_ is the normalized eigenvalue of the fabric tensor corresponding to the specific orthotropic plane. The modulus-BVF-fabric relationship was introduced by Garcia et al. [49]. The yield strain, 1.04%, is in accordance with the study of Bayrakhtar et al. [50]. The corresponding yield stress (*σ*_*y*_) was determined based on a 0.2% offset rule. Ultimate stress was set to 1.1*σ*_*y*_ in accordance with the study of Helgason et al. [53]. (b) A compressive load of *f*_*c*_ = 1.09 MPa was evenly distributed over the estimated nucleus pulposus contact area of the FE models. The models were constrained against displacement in all directions on the inferior side of the vertebrae.

Heterogeneous, orthotropic material constants were mapped to the bone elements of the FE meshes based on approach introduced by Zysset et al. [48]. The principal material directions of the elements were assumed to be aligned with the fabric tensor of the elements. Orthotropic elastic properties then were assigned for each element using a relationship based on density and fabric [49]. The material response was assumed to be non-linear with a yield strain in compression of 1.04%, in agreement with the study of [50] (Fig 1). Tension-compression symmetry in the mechanical properties was assumed. A pressure of 1.09 MPa was applied on the superior end-plate, uniformly distributed over the nucleus pulposus area, occupying 43% of the disc area [51] as shown in Fig 1. The inferior end-plate of the FE models was constrained against translation in all directions. The rationale of this was to mimic a high nucleus pressure during moderate daily activities, e.g. as observed in a healthy male subject when lifting a 20 kg weight and holding it close to the body [52].

Failure in the bone elements was assumed to occur if principal strains in tension (*ε*_*b*,*t*_) or compression (*ε*_*b*,*c*_) exceeded 1.5% and -2.0% respectively. The factor of fracture risk (*FFR*) was computed for each element (*i*) as the maximum of the ratio between the element principal strains (*ε*_*1*,*i*_ or *ε*_*3*,*i*_) and the corresponding principal strain threshold (*ε*_*b*,*t*_ or *ε*_*b*,*c*_) i.e.:

$${FFR}_{pre,i}\;=\;max(\frac{\varepsilon_{1,i}}{\varepsilon_{b,t}},\frac{\varepsilon_{3,i}}{\varepsilon_{b,c}})$$ (1)

### FE simulations—cement flow

After calculating the pre-augmentation fracture risk, flow simulations were carried out to simulate the distribution of cement within the specimens during vertebroplasty using different cannula placements, cement types and cement volumes. To this end the local heterogeneous hydraulic permeability of the cancellous bone was predicted by the FE bone volume fraction (*BVF*), the degree of anisotropy (*DA*) and the trabecular spacing (*Tb*.*Sp*) [54]. The flow behavior was governed by a mixed-boundary Darcy formulation [55]. The rheology of the bone cements was experimentally determined in a plate rheometer setup [56]. The deformation rate of a fluid that is infiltrating a porous medium such as the cancellous bone cannot be estimated or predicted at the continuum length scale, however it is an important determinant of the fluid rheology. Therefore, a numerical upscaling scheme was applied to relate the pore-scale viscosity of the cement, which is governed by the fluid deformation rate, to the apparent or Darcy viscosity at the continuum length scale [56]. In the simulations the infiltration of the vertebral body and displacement of the bone marrow by the virtually injected cement, i.e. the multicomponent flow of the bone cement and marrow was simulated. The cement injections were virtually performed at 50 randomly chosen locations within each of the FE models. The assumed locations of the injection device cannula were kept consistent according to the mesh morphing procedure across all four vertebrae specimens. Simulations were carried out for ten different clinically relevant cement volumes (*V* = 1, 2, 3, 4, 5, 6, 7, 8, 9, 10 mL).

### FE simulations—post-augmentation fracture risk

After the flow simulations, the material properties of the augmented elements in the FE models were mapped using the lookup Table approach introduced by Helgason et al. [57]. The rule of mixture in the present study was extended to the rule of mixture in Helgason et al. [57], to take into account that some elements of the FE meshes have their pore space only partially filled after the flow simulations. A reduction factor, *ξ* = 10%, was introduced to capture the fact that PMMA has lower strength at body temperature than when tested at room temperature [58]. The extended rule of mixture used in the present study was thus:

$$\sigma_{i}\;=\;\Phi_{j}\sigma_{UL}(\eta_{i},{BVF}_{i},\varepsilon_{i},\xi )+(1-\Phi_{j})\sigma_{LL}(\eta_{i},{BVF}_{i},\varepsilon_{i},\xi )\;=\;{c\Phi }_{j}({CVF}_{i}(1-\xi )\sigma_{c,j}+{BVF}_{i}\sigma_{b})+(1-{c\Phi }_{j})(\sigma_{LL,b}(\eta_{i},{BVF}_{i},\varepsilon_{i},\xi )+c(1-\xi )\sigma_{LL,c}(\eta_{i},{BVF}_{i},\varepsilon_{i},\xi ))$$ (2)

Where:

*i*: refers to the element number

*j*: refers to the cement type (see Table 2).

*BVF*_*i*_: bone volume fraction

*CVF*_*i*_: cement volume fraction

*η*_*i*_ = anisotropy ratio

*Φ*_*j*_ = cement specific rule of mixture constant (see Table 2).

*CVF*_*i*_∙(*1-ξ*)∙*σ*_*c*,*j*_ + *BVF*_*i*_∙*σ*_*b*_: is an approximation for the temperature adjusted upper limit response (*σ*_*UL*_) for an augmented element where *σ*_*c*,*j*_ and *σ*_*b*_ are the uniaxial stress response of a given cement (*j*) and the tissue level properties of bone respectively.

*c* = *CVF*_*i*_/(*1-BVF*_*i*_) is a constant that adjusts the uniaxial stress response of the augmented elements to take incomplete augmentation of an element after flow simulations into account. If the pore space is completely filled after the flow simulations *CVF* is equal to the pore space = *1-BVF* and thus *c = 1*.

**Table 2:** Properties related to the mechanical response of the PMMA cements used in present study. *E*: Modulus of elasticity; *Φ*: rule of mixture constant. The cements were selected from a range of commercial products (Simplex P; Stryker Howmedica Osteonics, Mahwah, NJ. Vertecem; Synthes GmbH, Solothurn, Switzerland. Active OS; Kyphon, Sunnyvale, CA), where the liquid phase was modified in our lab, in some cases, for the purpose of getting a wide range of stiffness for the cements used in the study; **A:** modified Simplex P. **B:** modified Simplex P. **C:** Modified Vertecem. **D:** Vertecem. **E:** Simplex P. **F:** Active OS.

Cement	*E* (MPa)	*ε*_*cem*_ (%)	*Φ* (-)
**A**	510	7.00	0.40
**B**	1434	7.10	0.40
**C**	2003	5.44	0.40
**D**	2697	5.10	0.29
**E**	2869	5.00	0.29
**F**	5193	3.93	0.15

The moduli of elasticity of the evaluated cements encompassed a range of *E* = 0.5 − 5.2 GPa, as defined in Table 2. The properties of the augmented elements were assumed to be orthotropic with the same principal material axes as the native bone only elements. The specimens were loaded and supported in the same manner as in the pre-augmentation simulation case and the post-augmentation risk of fracture for the augmented elements was calculated using:

$${FFR}_{post,i}\;=\;max(\frac{\varepsilon_{1,i}}{\varepsilon_{comp,t}},\frac{\varepsilon_{3,i}}{\varepsilon_{comp,c}})$$ (3)

Where *ε*_*comp*,*t*_ and *ε*_*comp*,*c*_ are the strain thresholds assumed to apply for each augmented element. These thresholds were defined with the following rule of mixture:

$$\varepsilon_{comp,t}\;=\;{CVF}_{i}∙\varepsilon_{cem}+{BVF}_{i}∙\varepsilon_{b,t}$$ (4)

$$\varepsilon_{comp,c}\;=\;{CVF}_{i}∙\varepsilon_{cem}+{BVF}_{i}∙\varepsilon_{b,c}$$ (5)

The *FFR*_*post*_ for bone bone elements was calculated using Eq 1.

### Post-processing of data

To quantify the effect of a given treatment strategy an organ fracture risk improvement factor (*OFRI*) was calculated according to:

$$OFRI\;=\;\frac{max_{\Omega }\left(FFR_{pre}\right)}{max_{\Omega }\left(FFR_{post}\right)}$$ (6)

Where *Ω* refers to all elements of the FE model and *FFR*_*pre*_ and *FFR*_*post*_ refer to the pre- and post-augmentation factor of fracture risk indicators, respectively. The *OFRI* is a measure of change in peak factor of fracture risk due to the treatment. A value for *OFRI* below unity indicates that a given treatment strategy increases the calculated risk of fracture compared to the *FFR*_*pre*_ risk but a value of *OFRI* above unity indicates a reduced calculated risk of fracture after the treatment.

For each specimen an N-way ANOVA (MATLAB R2012b, The Mathworks, Natick, USA), with the study parameters as the analysis factors, was performed to identify the effect of the treatments. p-values for the linear, two- and three-factor interactions were computed. p < 0.05 was considered as statistically significant.

## Results

**Fig 2:**
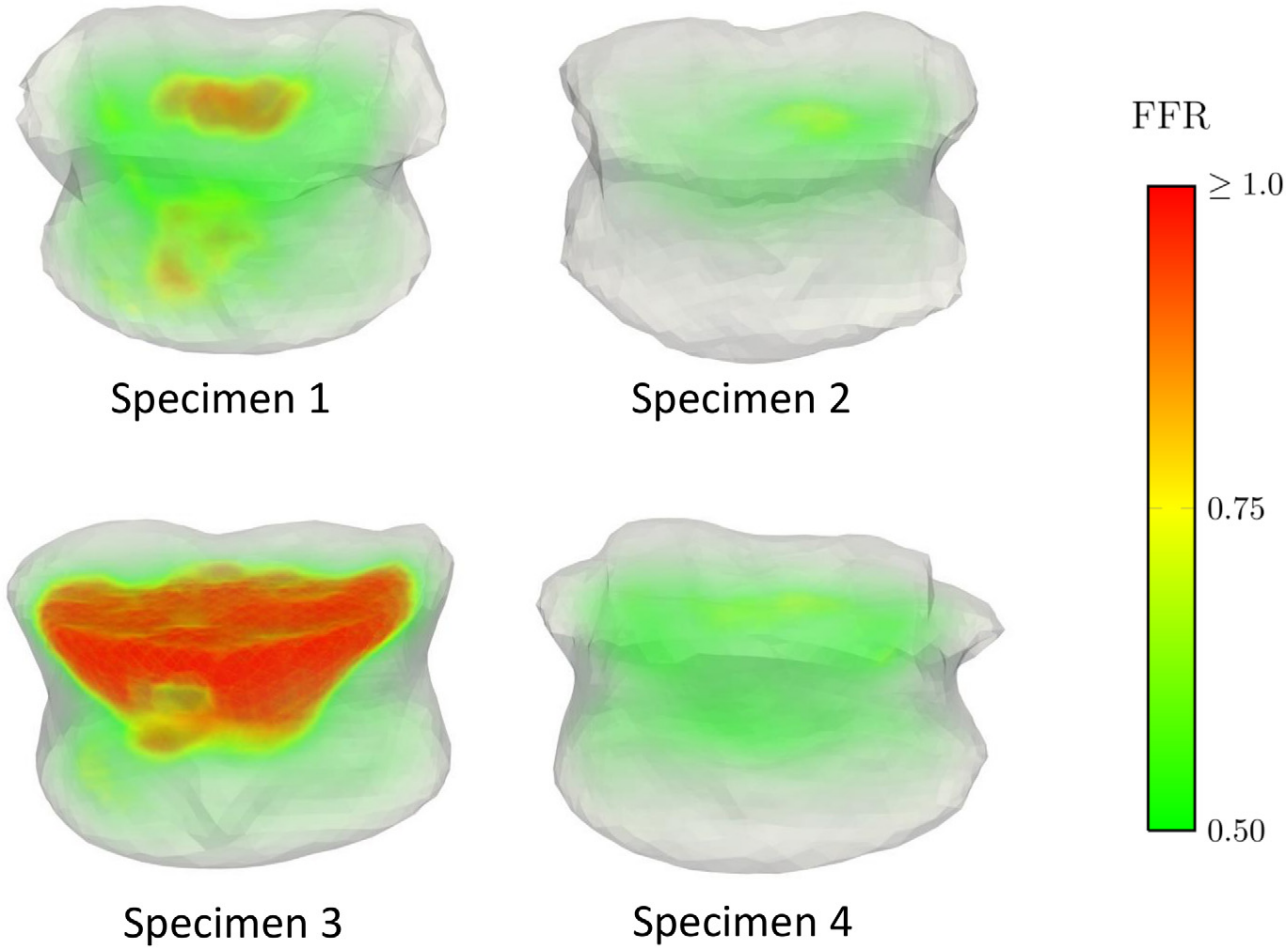
Pre-treatment initial factor of fracture risk (*FFR*_*pre*_). Distribution according to FE simulations for all of the specimens.

**Fig 3:**
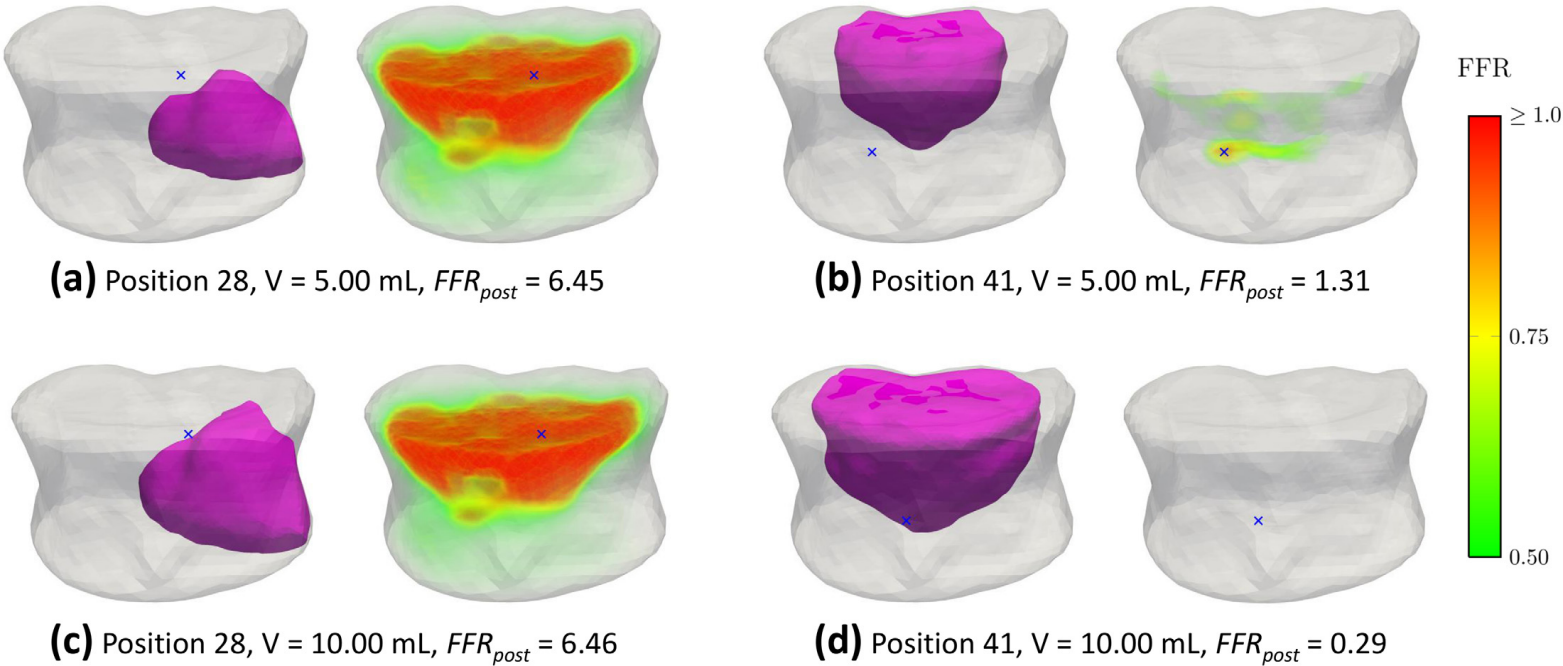
Comparison of pre- and post-augmentation fracture risk. The local change of the fracture risk following two treatment strategies and cannula placement trials with cement type F. The blue markers indicate the location of maximum fracture risk occurrence. (a) The asymmetric cement placement leads to a detrimental effect with a cement volume of *V* = 5.00 mL and even a large cement volume (*V* = 10.0 mL) is incapable of preventing a fracture (c). (b) A more centrally placed cement volume of *V* = 5.00 mL is nearly adequate to prevent the organ from overloading. (d) Injecting a volume of *V* = 10.0 mL prevents the organ from overloading.

**Fig 4:**
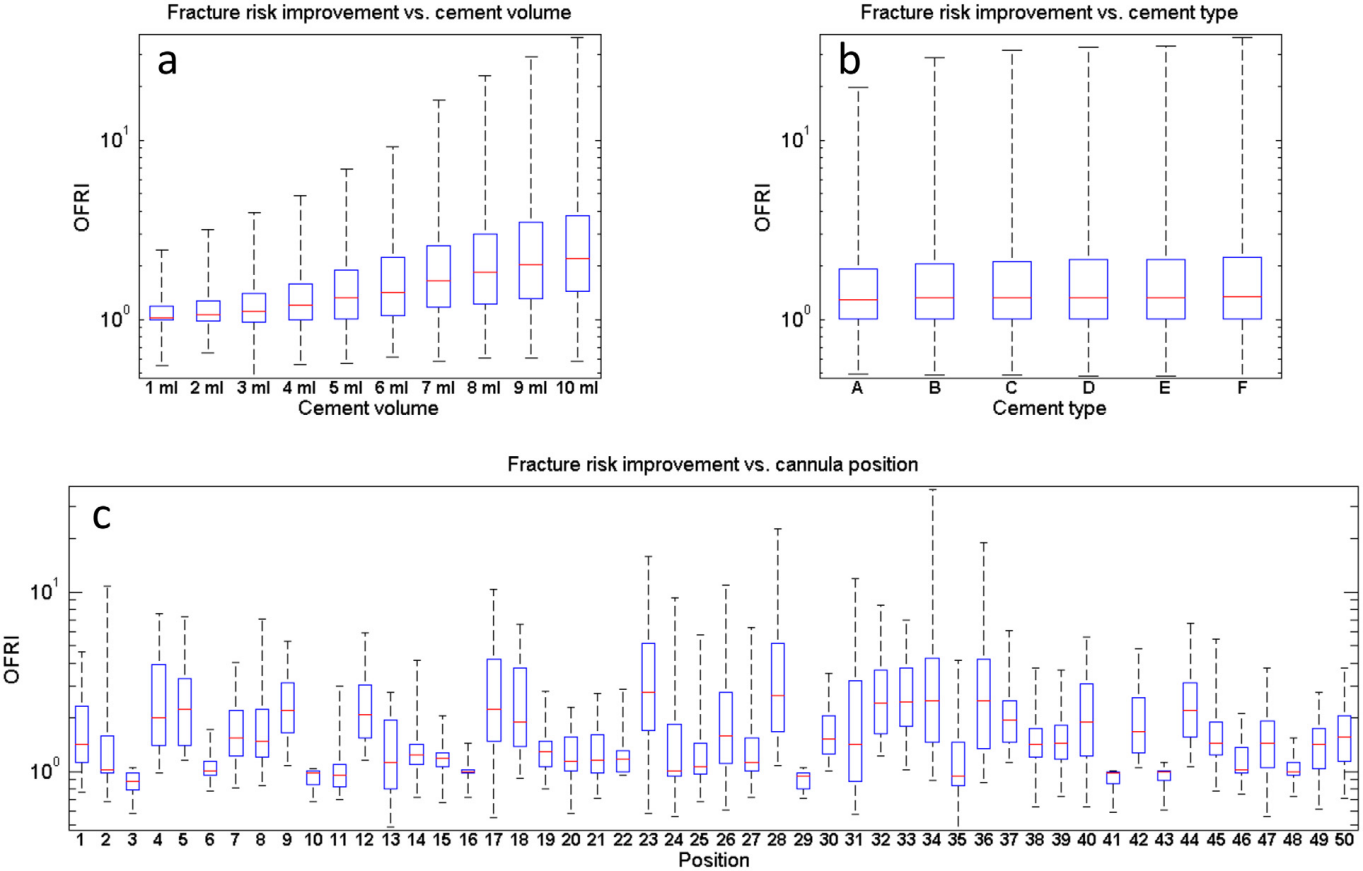
Post-augmentation fracture risk. The organ fracture risk improvement as a function of the injection volume (a), the cement type (b) and cannula position (c). The cannula position explains 47.2% of the variation in fracture risk reduction after treatment, and also highlights that a poorly planned intervention can even have a detrimental effect. The treatment outcome is also strongly related to cement volume (22.8% of the variability). The cement type only has a marginal influence on the overall effect (0.2% of the variability).

The local distribution of *FFR*_*pre*_ for all the specimens is illustrated in Fig 2. All of the specimens have a maximum predicted *FFR*_*pre*_ higher than one (Table 3) indicating that fracture is predicted for all the specimens prior to the augmentation. Sample simulated cement spreading patterns and local *FFR*_*post*_ -distributions for two cannula placement trials and two injection volumes are shown in Fig 3. The results for predicted values of *FFR* for pre and post-augmentation risk of fracture, as well as fracture risk improvement indicator (*OFRI*), are listed in Table 3. The organ fracture risk improvement indicator (*OFRI*) ranged from 0.47 to 37.23 depending on specimen, position, injected volume and cement type (Fig 4). Out of all treatments across all specimens, 76.6% resulted in an improved predicted fracture risk (*OFRI* > 1), 1.5% in no-effect (*OFRI* = 1) and 21.8% in a negative effect (*OFRI* < 1). Approximately 40% of the treatment strategies resulted in *FFR*_*post*_ being lower than unity but this number varied considerably between specimens, being 21, 74, 3, and 68% for specimens 1 to 4, respectively.

**Table 3:** Pre- and post-augmentation risk for fracture (*FFR*_*pre*_ and *FFR*_*post*_) and fracture risk improvement indicator (*OFRI*).

Specimen	*FFR*_*pre*_	*FFR*_*post*_	*OFRI*
1	1.95	0.29–3.43	0.57–6.76
2	1.08	0.03–2.28	0.47–37.23
3	6.41	0.29–11.60	0.55–22.44
4	1.06	0.17–1.79	0.59–6.21

The N-way ANOVA indicated that 9.9% of the variance in *OFRI* is explained by specimen variability, 22.8% of the variance in *OFRI* is explained by cement volume, 0.2% of the variance in *OFRI* explained by cement type, 47.2% of the variance in *OFRI* explained by cannula position and 19.9% of the variance is unexplained. All the variability was found to be significant (p < 0.05).

## Discussion

The aim of this study was to systematically investigate potential percutaneous vertebroplasty strategies in an in-silico environment to establish whether the treatment is efficient from a biomechanical point of view, and what the important determinants of a positive outcome are.

The findings of this study demonstrate that the outcome of vertebroplasty from a biomechanical perspective is complex and dependent on multiple factors. We found that PVP considerably improved the strength and reduced the calculated fracture risk in 76.8% of the treatment strategies simulated, but that the largest contributor to variation in outcome is associated with the cannula placement. However, a treatment can increase the predicted fracture risk and highlights that PVP can even have a detrimental effect. This has also been reported by Higgins et al. [36]. Generally, a good treatment outcome is associated with a cannula placed close to the central aspect of the vertebral body. This finding is consistent with the conclusions of other studies, where asymmetric placement of cement has been shown to reduce the biomechanical stability of osteoporotic vertebra [24, 31].

Biomechanical studies have reported that small amounts of cement resulted in a statistically significant increase in terms of strength [25, 36, 41] or stiffness [31, 35], compared to untreated vertebrae. Our data support this hypothesis only partially. For all cement volumes, we found treatment strategies that affected fracture risk both positively and negatively; however, with a clear trend towards higher cement volumes resulting in lower predicted fracture risk. Although our data suggests that large injection volumes are beneficial, it has been proposed that subsequent vertebral compression fractures of adjacent or remote levels may be caused by the increased stiffness of the treated vertebrae as a result of the amount of cement injected [28–30, 59] and are biomechanically not optimal [31]. The in-silico models in the present study however, are not capable of predicting the fracture risk in adjacent or remote levels. Therefore, this risk is not included in our models, but although indicated, large cement volumes do not have to be implicitly beneficial. Additionally, other biomechanical [60] and clinical studies [17, 61] found no statistically significant correlation between cement volume and pain relief. Therefore, large cement volumes should not be injected on a routine basis. Furthermore, the potential biomechanical benefits of increased cement volumes should be considered against the risks of cement or marrow extravasation.

We found that only 40% of the treatment strategies across all specimens resulted in a post treatment fracture risk (*FFR*_*post*_) below unity. However, the absolute values of the *FFR* factor have to be interpreted with care because the current in-silico prediction of the fracture risk is based on a single simplified load case. In addition, it is unknown to which extent bone can be loaded without causing pain, therefore we defined relatively conservative strain thresholds for defining failure from a biomechanical point of view. The specimen variability in the predicted fracture risk after treatment is of interest. Only 3% of the treatment strategies resulted in a *FFR*_*post*_ factor below unity for specimen 3 but 67% for specimen 4 from the same donor. This could, in our opinion, further support the conclusion that the treatment of osteoporotic vertebrae needs to be carefully planned in order to capture all the weaknesses when augmenting the cancellous compartment. These results thus only conditionally support the statement that small cement volumes on the order of 30% (4–8 mL, [60]) are sufficient to achieve good outcomes [17, 25, 31, 35] because by studying our data in more detail we found e.g. that no cannula positions would produce a *FFR*_*post*_ below unity with a cement volume smaller than 6 ml for specimen 3.

It has been reported that highly osteoporotic patients may receive the least amount of improvement in vertebral mechanical strength after vertebroplasty [62] and PVP is unnecessary for a subgroup of patients with osteoporotic fractures [9]. Moreover, the data of this study highlight the sensitivity of the mechanical response on the treatment parameters [31, 61]; critics would say that pain relief by vertebroplasty can only be achieved by chance, whereas optimists argue that the uncertainty can be eliminated by optimization techniques. The computational model presented in this study is a first attempt to solve this optimization problem by considering the individual patient’s local bone condition prior to treatment and investigating different treatment strategies with the novelty of a rapid, patient-specific prediction of the ultimate bone strength and cement spreading pattern. The advantage and importance of a patient-specific procedure to maximize the mechanical benefits while minimizing the risk of complications have been pointed out by Higgins et al. [36]. Furthermore, the cement spreading pattern is said to be influential on the stiffening and strengthening of the vertebral body [24, 31]. The FE model incorporates a simplified but patient-specific mechanical model of the vertebra, with only compressive loading and prophylactic vertebroplasty simulated. This has the advantage that multiple treatment scenarios can be rapidly computed and compared, which is considered valid and well-accepted for the accurate prediction of compressive strength and fracture risk [39, 63]. It is recommended, however, that a conclusive assessment of treatment effectiveness be verified through the simulation of functional spinal units (FSUs) including fractured vertebrae in order to assess fracture risk at adjacent or remote levels, incorporating spectra of multi-axial loading and age-related daily activities.

There are several limitations in our study that need to be addressed. First, we are using intact specimens in our simulations, whereas PVP is generally performed to treat fractured vertebrae in the clinic. The FE models would thus only directly simulate the prophylactic augmentation of vertebrae at risk, which is a potential future development of clinical procedures. Nevertheless, we believe that carrying this study out on fractured specimens would only further strengthen our main conclusion from this work, which is that treatment needs to be carefully planned in order to have a biomechanical effect, as fractured vertebrae demonstrate a high degree of heterogeneity in the local bone properties. We believe that the added complexity of fracture patterns in the specimens would most likely contribute to increase the number of unsuccessful treatment outcomes. Second, we are only simulating unipedicular injection of cement but bi-pedicular injection procedures are also used in clinical practice. However, the goal of the present study was not to find out which treatment strategy to use, which could be subject specific anyway, but rather to determine which treatment parameters the outcome is most sensitive to.

In summary our results suggest that: (a) the outcome and effectiveness of vertebroplasty is strongly determined the patient’s bone condition prior to treatment, which defines the baseline and the amount of mechanical stabilization required for the prevention of subsequent fractures; (b) not all treatment strategies are suitable for accomplishing this mechanical stabilization; and (c) the risk of treatment complications (subsequent fractures in adjacent or remote levels, cement leakage) might be a limiting factor for a treatment success. Whilst the latter has been recognized and thoroughly discussed in literature, until now the first two aspects were not given enough attention and might at least partially explain the controversial conclusions drawn from the randomized controlled trials that support or question the effectiveness of vertebroplasty.

## Supporting Information

S1 Data Risk of fracture results. This Matlab file contains the predicted risk of fracture before and after augmentation for all of the 12000 simulations cases of the study.
(MAT)
